# Supplementary material for: Increased VEGF‐A promotes multiple distinct aging diseases of the eye through shared pathomechanisms
Source: EMBO Mol Med. 2016 Feb 24;8(3):208–31. doi: 10.15252/emmm.201505613 (PMC4772957; doi:10.15252/emmm.201505613)
Supplement: Supplementary file 1 — Appendix [file EMMM-8-208-s001.pdf]

APPENDIX:

1. Appendix Figure legends S1 to S6 and Appendix Table S1.

2. Appendix Figures S1 to S6

APPENDIX FIGURE LEGENDS:

Appendix Figure S1: Quantification of cataract formation in VEGF-A^{hyper} mice and WT littermate mice, as well as mouse groups lacking Nlrp3 or Il1r1.

A. Calculation of p-values in mouse groups >18 months of age with +3 cataracts. P-values were calculated with a two-tailed unpaired student's t-test.

B. Cataracts were graded from 0 (no cataract) to +1 (mild), +2 (moderate) or +3 (severe) cataract. Values indicate mean ± SEM in groups of mice >18 months of age (N indicates number of mice in these groups that were available for analysis).

C. Tables show absolute numbers and percentile numbers of cataracts for each grading in each age-group of VEGF-A^{hyper} mice, WT mice, Nlrp3$^{-/-}$ mice, VEGF-A^{hyper}/Nlrp3$^{-/-}$ mice, Il1r1$^{-/-}$ mice, and VEGF-A^{hyper}/Il1r1$^{-/-}$ mice.

Appendix Figure S2: AMD-like pathologies in aged VEGF-A^{hyper} mice.

A.-D. Electron microscopy images of 21-months old wild-type control littermate mice (WT) and age-matched VEGF-A^{hyper} mice. WT littermate mice show a normal RPE cell layer and Bruch's membrane (*). Photoreceptor outer segments (OS) interdigitate with RPE apical villi. In age-matched VEGF-A^{hyper} mice there is accumulation of sub-RPE basal laminar-like deposits that are associated with degenerative changes of the RPE (including RPE atrophy and loss of pigment granules) and photoreceptor degeneration.

White double arrows indicate RPE. Black double arrows indicate sub-RPE deposits.

Scale bars for A, B and D are 500nm and for C 2µm.

E. Light microscopy image of a WT control littermate mouse eye (28-months old). Asterisk (*) indicates normal Bruch's membrane. Normal age-appropriate RPE cell layer without atrophy or pigment loss is seen. Photoreceptors show no degenerative changes.

F.-H. Thickened Bruch's membrane (*) with sub-RPE deposits that protrude into the RPE cell plane (white arrows). These deposits are separated from the photoreceptors by a layer of abnormal RPE cells (black arrows). Degenerative changes of the adjacent photoreceptors are observed (red arrows). CNV vessel containing erythrocytes is separated from the photoreceptor layer by RPE cells (yellow arrow in H.). Scale bars 20µm.

Chor: choroid; RPE: retinal pigment epithelium; ONL: outer nuclear layer; OS: photoreceptor outer segments; *: Bruch's membrane.

Appendix Figure S3: Electron microscopy shows degenerative changes of the RPE, Bruch's membrane and photoreceptors in aged VEGF-A^{hyper} mice.

A.-J. Representative electron microscopy images of the choroid/RPE/photoreceptor interface in 21-months old VEGF-A^{hyper} mice show Bruch's membrane thickening (*), sub-RPE deposit formation (white arrows), RPE degeneration (with atypical pigment granules and loss of pigment, as well as RPE atrophy and degenerative changes) (black arrows), and photoreceptor abnormalities (red arrows). Endothelial fenestrations of the choriocapillaris are maintained in these aged VEGF-A^{hyper} mice (yellow arrow; seen in J.).

Scale bars: A, B and E 600nm; C 2.1μm; D, F, G and I 2μm; H 450nm; and J 90nm.

Appendix Figure S4: Conditional inactivation of Flk1 in the RPE inhibits CNV lesion formation in VEGF-A^{hyper} mice.

Choroidal flat mount staining of a 8-months old Vmd2Cre$^{+/WT}$Flk1$^{fl/fl}$ VEGF-A^{hyper} mouse shows nuclear β-gal staining (green), representing VEGF-A expression in the RPE. Uniform nuclear Cre staining (red) is observed in RPE cells of Vmd2Cre$^{+/WT}$Flk1$^{fl/fl}$ VEGF-A^{hyper} mice. Phalloidin staining (white) shows no CNV lesion formation in this eye (upper panel). Nuclear co-localization of Cre and β-gal staining shown in higher magnification images from this flat mount staining (lower panel). Scale bars: upper panel 200 μm, lower panel 50μm.

Appendix Figure S5: CNV lesion formation is regulated by NLRP3 inflammasome components in VEGF-A^{hyper} mice.

A. Square root transformation of CNV lesion numbers/eye in each experimental mouse group. Casp1/Casp11, Nlrp3 or Il1r1 deficiency results in significantly fewer CNV lesions.

B. Log transformation of average CNV lesion area/eye in each experimental mouse group. Lack of Tlr2 or Casp1/Casp11 significantly reduces CNV lesion size, while reduced autophagy moderately increases CNV size.

Graphs indicate values for each individual mouse. Mean ± SEM is shown. P-values are compared with the VEGF-A^{hyper} mouse group in each graph. Absolute numbers of mice per group are indicated in ( ). P-values were calculated with a two-tailed unpaired student’s t-test. Separately, p-values are shown between VEGF-

A^{hyper}/Casp1$^{-/-}$/Casp11$^{-/-}$ and VEGF-A^{hyper}/Nlrp3$^{-/-}$ or VEGF-A^{hyper}/Il1r1$^{-/-}$ mouse groups. WT mice developed no CNV lesions.

Appendix Figure S6: Nlrp3^{A350VneoR} mice, referred to as Nlrp3$^{-/-}$ mice, lack Nlrp3 transcripts.

RT-PCR using primers that span exon 2 and exon 3 of Nlrp3 show expression of Nlrp3 in spleen of WT or VEGF-A^{hyper} mice, but not in mice with the Nlrp3^{A350VneoR} allele (Nlrp3$^{-/-}$ mice), which have a *neoR* cassette in intron 2 of the Nlrp3 gene. 36B4: housekeeping gene control.

Appendix Table S1:

Semiquantitative RT-PCR primers used in this study:

Primer Name	Primer Sequence
m36B4 up	TCACTGTGCCAGCTCAGAAC
m36B4 dw	AATTTCAATGGTGCCTCTGG
mCatalase up	TGAGAAGCCTAAGAACGCAATTC
mCatalase dw	CCCTTCGCAGCCATGTG
mGP91 phox up	TTGGGTCAGCACTGGCTCTG
mGP91 phox dw	TGGCGGTGTGCAGTGCTATC
mGPx1 up	CGCTTTCGTACCATCGACATC
mGPx1 dw	GGGCCGCCTTAGGAGTTG
mIL1b up	GCTTCCAAACCTTTGACCTG
mIL1b dw	CTGTTGTTTCCCAGGAAGAC
mIL18 up	CAGGCCTGACATCTTCTGCAA
mIL18 dw	CTGACATGGCAGCCATTGT
mNLRP3 up	GTGGATGGGTTTGCTGGGAT
mNLRP3 dw	TCTTGCACACTGGTGGGTTT
mSOD1 up	GTGATTGGGATTGCGCAGTA
mSOD1 dw	TGGTTTGAGGGTAGCAGATGAGT
total VEGFA R (VEGF A rev)	CATCAGCGGCACACAGGACGG
total isoform common (VEGF A for)	CCCACGACAGAAGGAGAGCAGAAGT

A

	p-values 18+ months groups
WT vs VEGF-A^{hyper}	<0.0001
Nlrp3$^{-/-}$ vs VEGF-A^{hyper}/Nlrp3$^{-/-}$	0.0002
WT vs Nlrp3$^{-/-}$	0.0363
VEGF-A^{hyper} vs VEGF-A^{hyper}/Nlrp3$^{-/-}$	0.6369
WT vs VEGF-A^{hyper}/Nlrp3$^{-/-}$	<0.0001

B

	MEAN ± SEM, N (18+ months old groups)
WT	0.7403 ± 0.07811 N=181
Nlrp3$^{-/-}$	0.0 ± 0.0 N=9
VEGF-A^{hyper}	2.037 ± 0.1733 N=54
VEGF-A^{hyper}/Nlrp3$^{-/-}$	1.893 ± 0.2589 N=28

C

absolute	WT				sum	VEGF-A^{hyper}				sum
	0	1	2	3		0	1	2	3	
0-3 months	82	0	0	1	83	47	3	0	0	50
3-6 months	18	1	0	0	19	9	0	0	2	11
6-9 months	17	0	0	1	18	6	3	2	4	15
9-12 months	44	2	1	0	47	15	4	3	15	37
12-18 months	31	6	1	0	38	5	7	6	21	39
18+ months	107	36	16	22	181	12	6	4	32	54
sum	299	45	18	24	386	94	23	15	74	206

%	WT				sum	VEGF-A^{hyper}				sum
	0	1	2	3		0	1	2	3	
0-3 months	98.80	0.00	0.00	1.20	100	94.00	6.00	0.00	0.00	100
3-6 months	94.74	5.26	0.00	0.00	100	81.82	0.00	0.00	18.18	100
6-9 months	94.44	0.00	0.00	5.56	100	40.00	20.00	13.33	26.67	100
9-12 months	93.62	4.26	2.13	0.00	100	40.54	10.81	8.11	40.54	100
12-18 months	81.58	15.79	2.63	0.00	100	12.82	17.95	15.38	53.85	100
18+ months	59.12	19.89	8.84	12.15	100	22.22	11.11	7.41	59.26	100

absolute	Nlrp3$^{-/-}$				sum	VEGF-A^{hyper}/Nlrp3$^{-/-}$				sum
	0	1	2	3		0	1	2	3	
0-3 months	56	0	0	0	56	59	0	0	0	59
3-6 months	36	0	0	3	39	20	0	0	1	21
6-9 months	9	1	0	0	10	17	1	1	4	23
9-12 months	20	3	0	0	23	22	2	1	4	29
12-18 months	8	0	1	0	9	13	2	1	8	24
18+ months	9	0	0	0	9	8	3	1	16	28
sum	138	4	1	3	146	139	8	4	33	184

%	Nlrp3$^{-/-}$				sum	VEGF-A^{hyper}/Nlrp3$^{-/-}$				sum
	0	1	2	3		0	1	2	3	
0-3 months	100.00	0.00	0.00	0.00	100	100.00	0.00	0.00	0.00	100
3-6 months	92.31	0.00	0.00	7.69	100	95.24	0.00	0.00	4.76	100
6-9 months	90.00	10.00	0.00	0.00	100	73.91	4.35	4.35	17.39	100
9-12 months	86.96	13.04	0.00	0.00	100	75.86	6.90	3.45	13.79	100
12-18 months	88.89	0.00	11.11	0.00	100	54.17	8.33	4.17	33.33	100
18+ months	100.00	0.00	0.00	0.00	100	28.57	10.71	3.57	57.14	100

absolute	Il1r1$^{-/-}$					VEGF-A^{hyper}/Il1r1$^{-/-}$				
	0	1	2	3	sum	0	1	2	3	sum
0-3 months	62	0	0	0	62	15	0	0	0	15
3-6 months	39	0	0	0	39	1	0	0	0	1
6-9 months	20	0	0	0	20	3	1	0	1	5
9-12 months	15	0	0	0	15	7	0	0	0	7
12-18 months	6	0	0	0	6	3	1	0	1	5
18+ months	17	1	0	1	19	6	4	0	1	11
sum	159	1	0	1	161	35	6	0	3	44

%	Il1r1$^{-/-}$					VEGF-A^{hyper}/Il1r1$^{-/-}$				
	0	1	2	3	sum	0	1	2	3	sum
0-3 months	100.00	0.00	0.00	0.00	100	100.00	0.00	0.00	0.00	100
3-6 months	100.00	0.00	0.00	0.00	100	100.00	0.00	0.00	0.00	100
6-9 months	100.00	0.00	0.00	0.00	100	60.00	20.00	0.00	20.00	100
9-12 months	100.00	0.00	0.00	0.00	100	100.00	0.00	0.00	0.00	100
12-18 months	100.00	0.00	0.00	0.00	100	60.00	20.00	0.00	20.00	100
18+ months	89.47	5.26	0.00	5.26	100	54.55	36.36	0.00	9.09	100

Appendix Figure S1

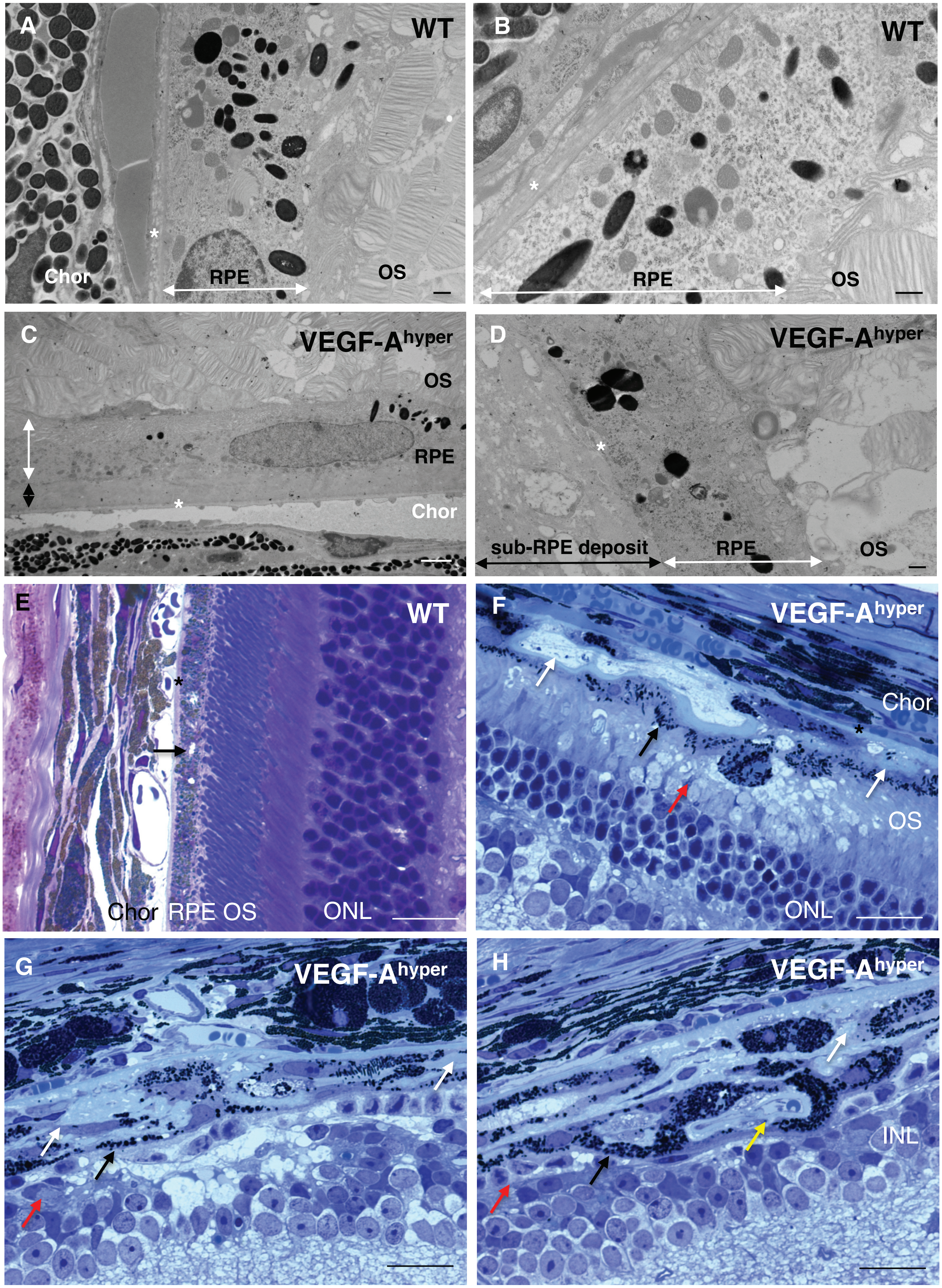

Appendix Figure S2

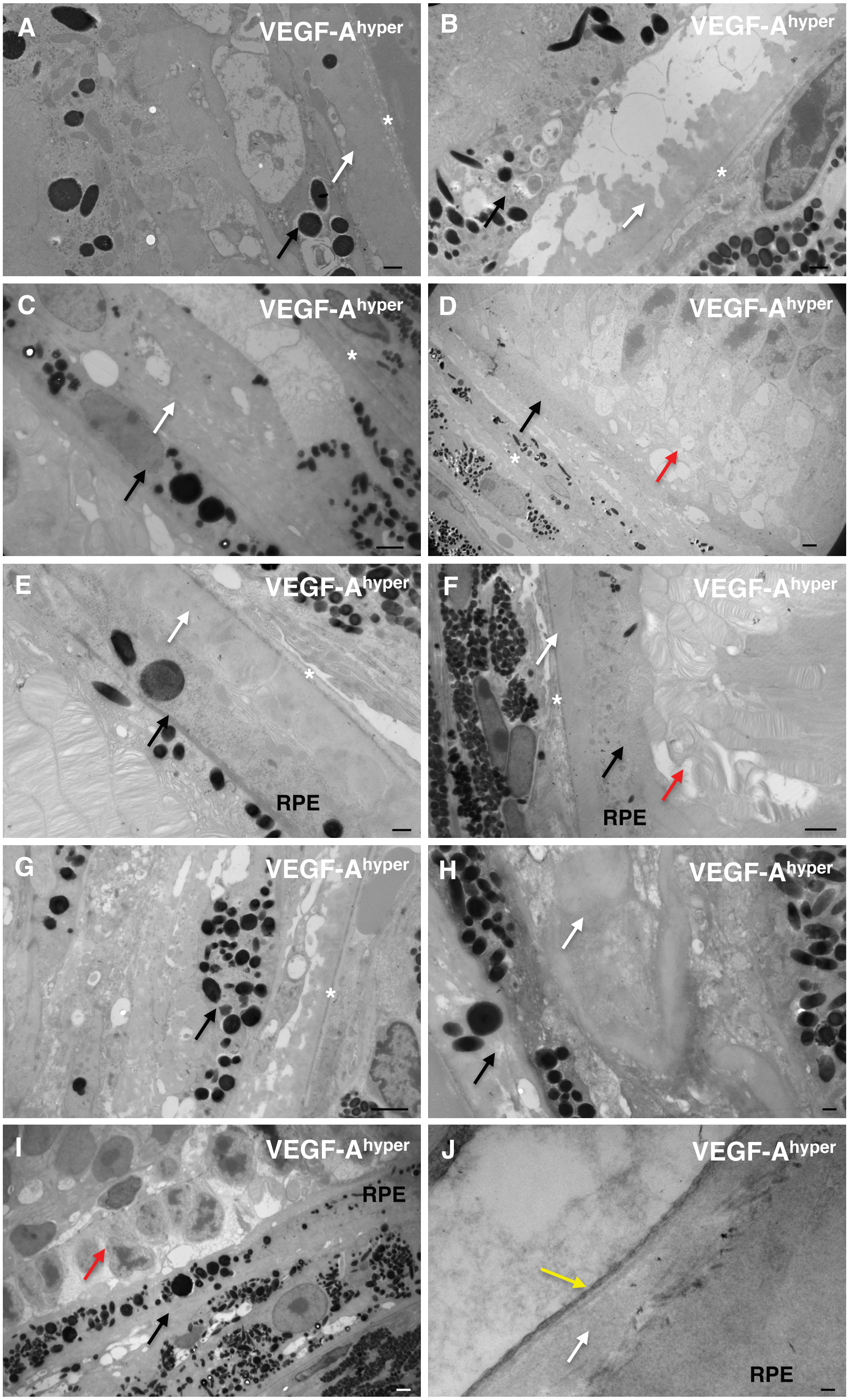

Appendix Figure S3

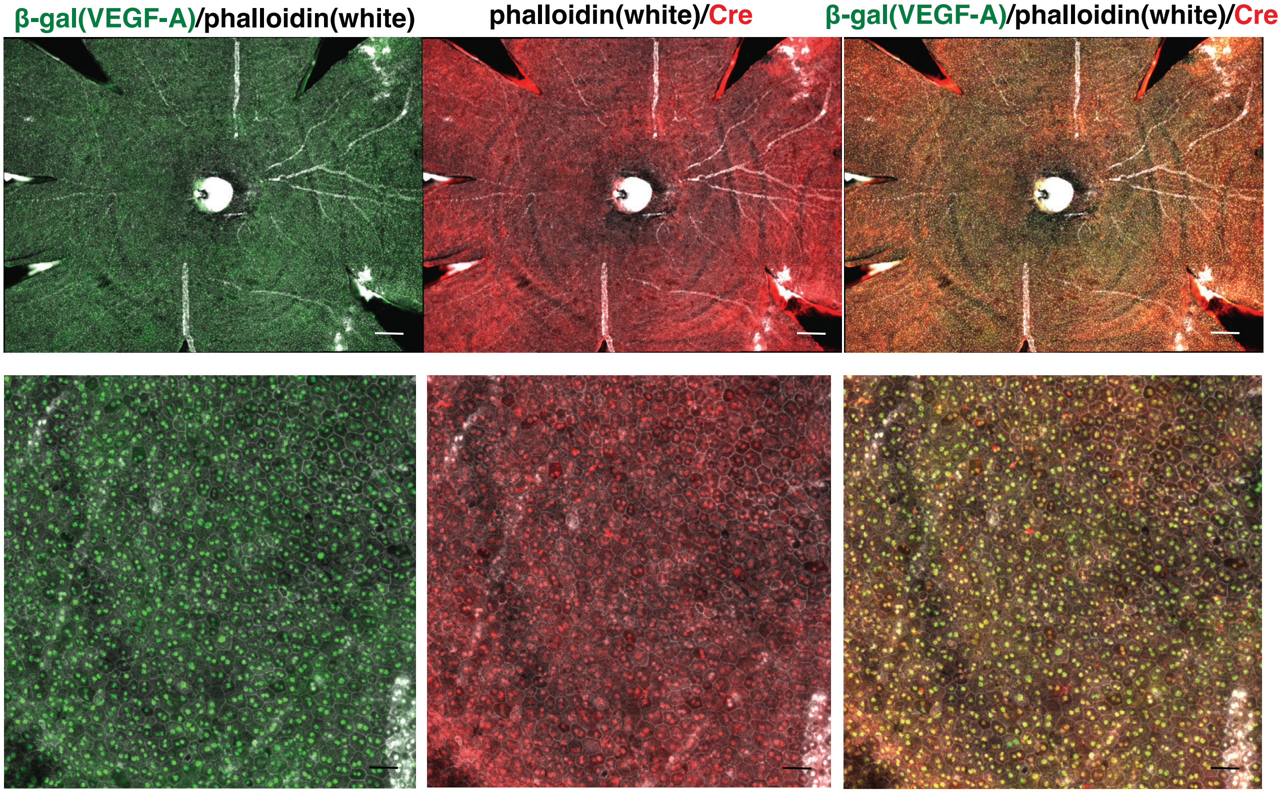

Appendix Figure S4

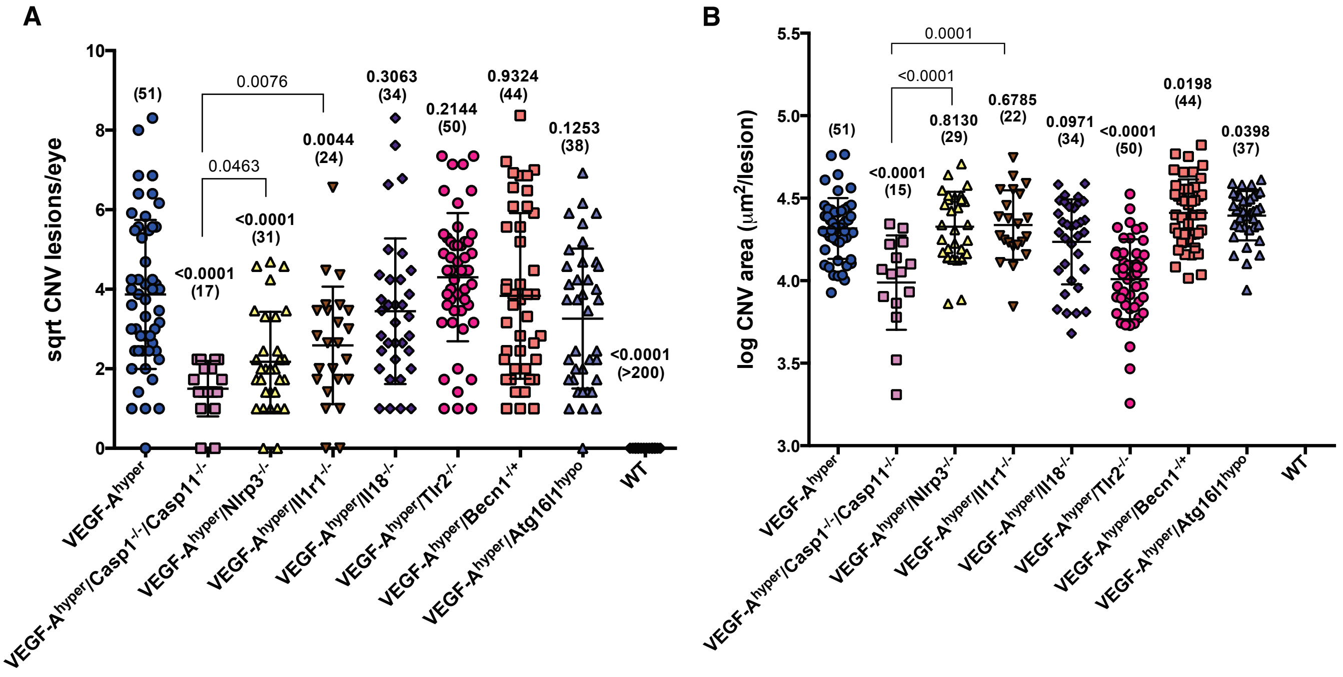

Appendix Figure S5

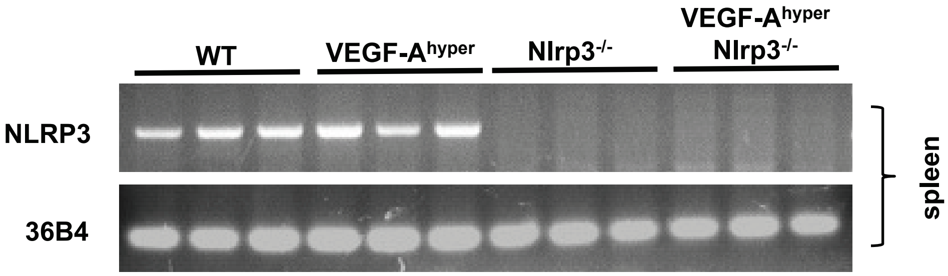

Appendix Figure S6
