# Supplementary material for: Increased VEGF‐A promotes multiple distinct aging diseases of the eye through shared pathomechanisms
Source: EMBO Mol Med. 2016 Feb 24;8(3):208–31. doi: 10.15252/emmm.201505613 (PMC4772957; doi:10.15252/emmm.201505613)
Supplement: Supplementary file 3 — Source Data for Figure 2 [file EMMM-8-208-s002.pdf]

Figure 2B, uncropped gels lens, RT-PCR

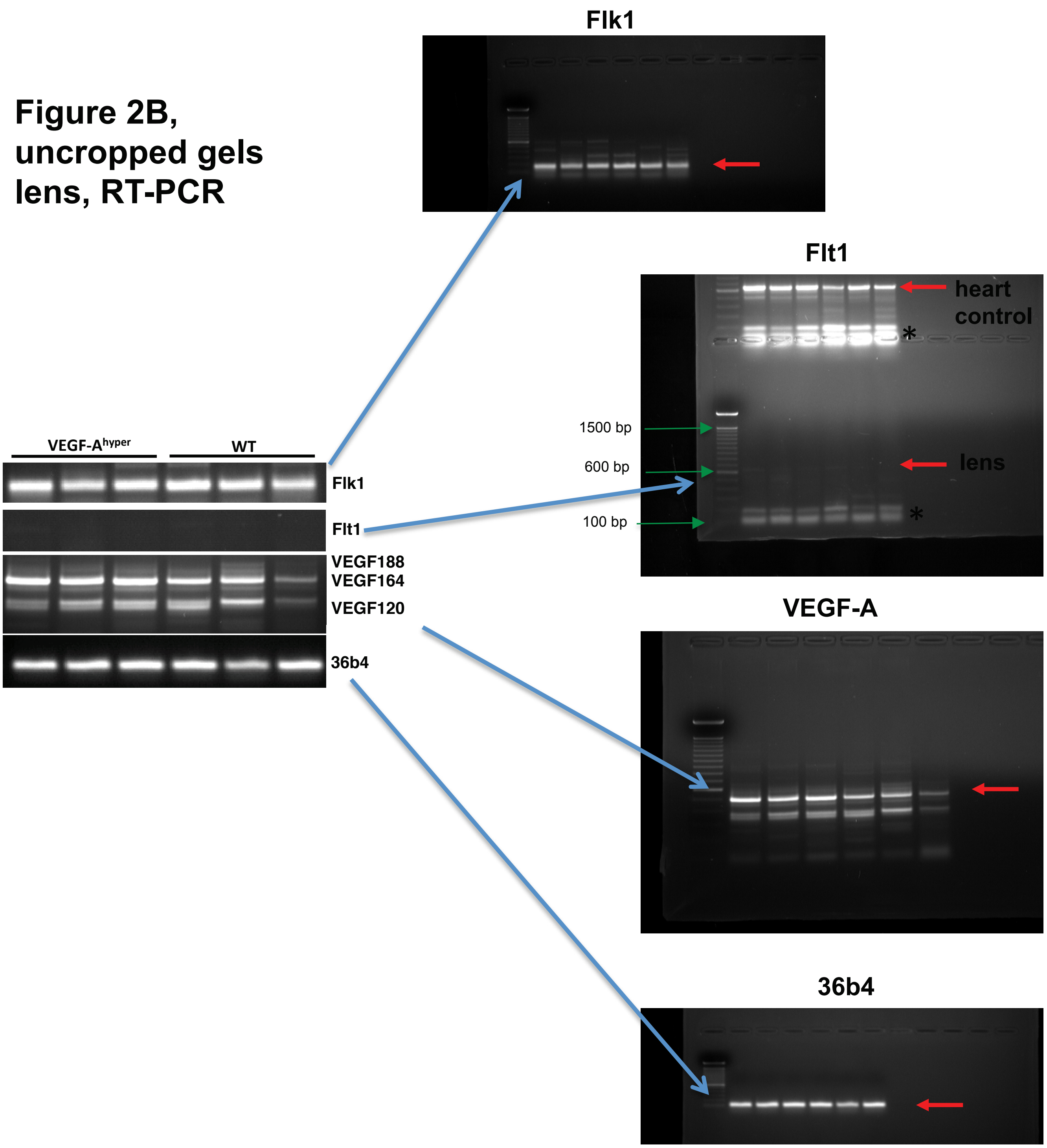

* background band

red arrows indicate expected bands (for all gels TrackIt 100bp DNA ladder was used (Life Technologies))
