# Supplementary figures and images for: Increased VEGF‐A promotes multiple distinct aging diseases of the eye through shared pathomechanisms
Source: EMBO Mol Med. 2016 Feb 24;8(3):208–31. doi: 10.15252/emmm.201505613 (PMC4772957; doi:10.15252/emmm.201505613)

Full unedited gel, Figure 3a

Lens  
ERK-P

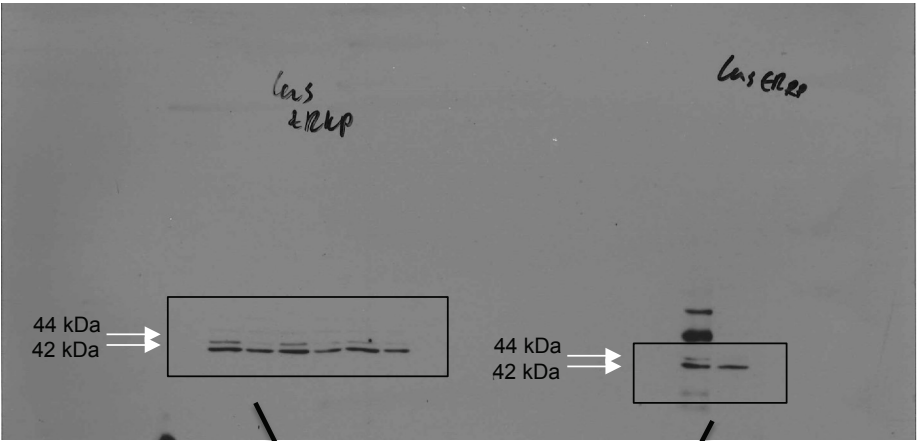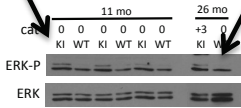

Lens  
Total ERK

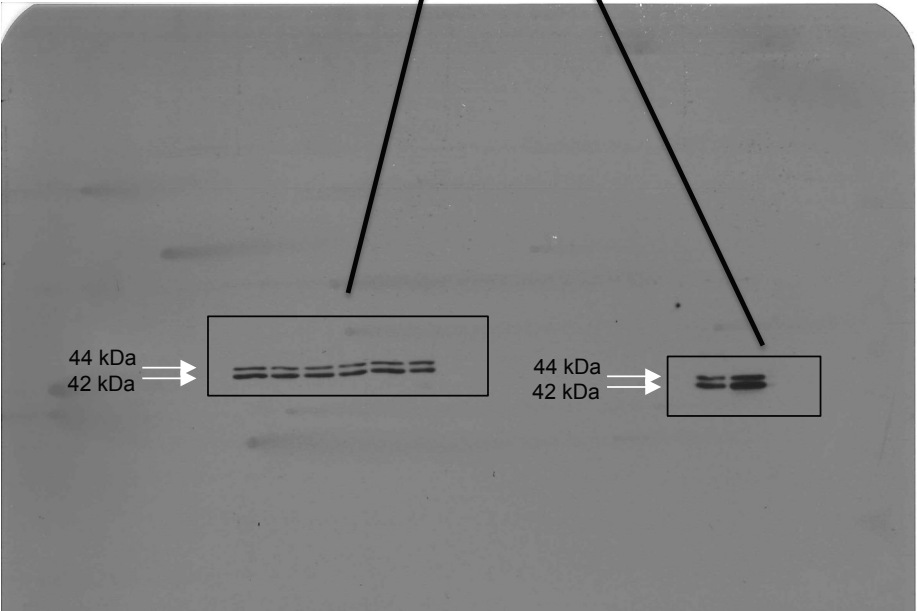

Supplement: Supplementary file 4 — Source Data for Figure 3 [file EMMM-8-208-s003.pdf]
